# Supplementary material for: Kidney Replacement Treatment in South-Western Italy (Campania): Population-Based Study on Gender and Residence Inequalities in Health Care Access
Source: J Clin Med. 2021 Jan 24;10(3):449. doi: 10.3390/jcm10030449 (PMC7865879; doi:10.3390/jcm10030449)

SUPPLEMENTAL

MATERIAL

Table S1 – Descriptive statistics.

Number of patients	11713
Male gender, %	61.5%
Age at entry in datafile, years	63.1±16.2 (0/100)
% with age < 18	1.2%
% with age 18-44	11.7%
% with age 45-64	35.0%
% with age 65-74	25.0%
% with age ≥ 75	27.2%
Local health authority of residence	
Napoli 1 centro	18.6%
Napoli 2 nord	16.9%
Napoli 3 sud	18.5%
Avellino	7.5%
Benevento	4.3%
Caserta	14.9%
Salerno	19.3%

Figure S1 – Prevalence of kidney replacement treatment, haemodialysis, peritoneal dialysis, and kidney transplant among residents of Campania region in year 2015: patients per million population (pmp) by age and gender (blue line for males and red line for females, respectively). KRT: kidney replacement treatment

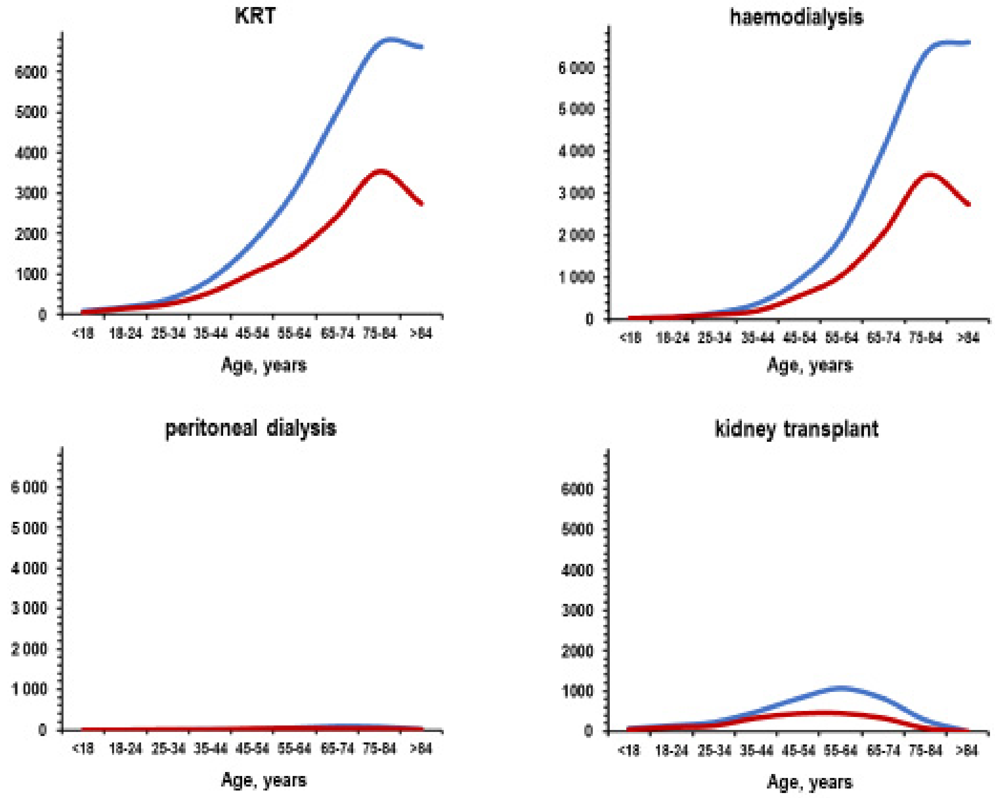

Figure S2 – Prevalence of kidney replacement treatment, haemodialysis, peritoneal dialysis, and functioning kidney transplant among residents of Campania region in year 2016: patients per million population (pmp) by age and gender (blue line for males and red line for females, respectively). KRT: kidney replacement treatment

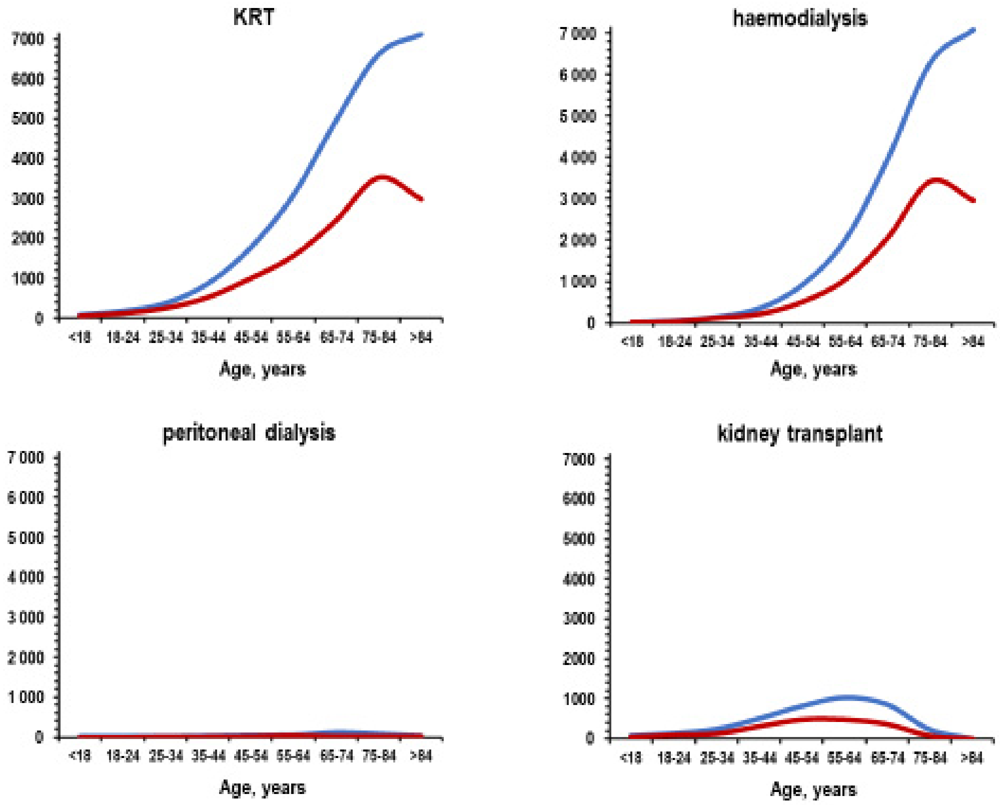

Figure S3 – Prevalence of kidney replacement treatment, haemodialysis, peritoneal dialysis, and functioning kidney transplant among residents of Campania region in year 2018: patients per million population (pmp) by age and gender (blue line for males and red line for females, respectively). KRT: kidney replacement treatment

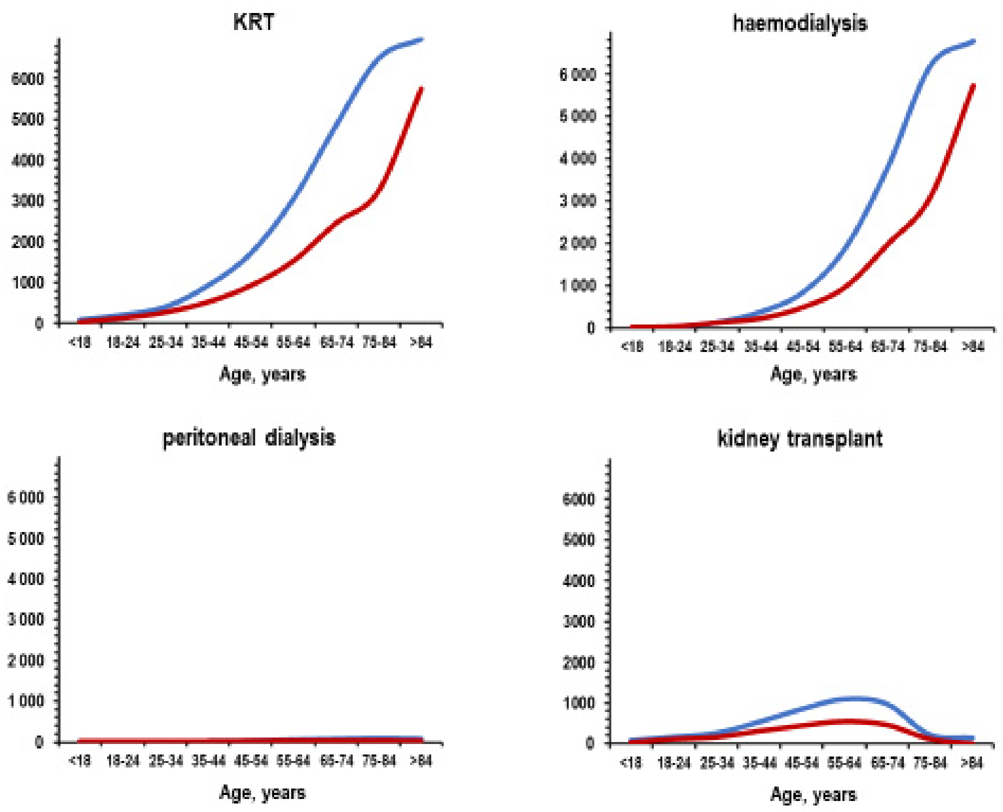

Figure S4 – Prevalence of peritoneal dialysis in the total of patients on dialysis from 2015 to 2018 among residents of Campania region: % patients by gender and by age at entry in datafile (blue line for males and red line for females, respectively).

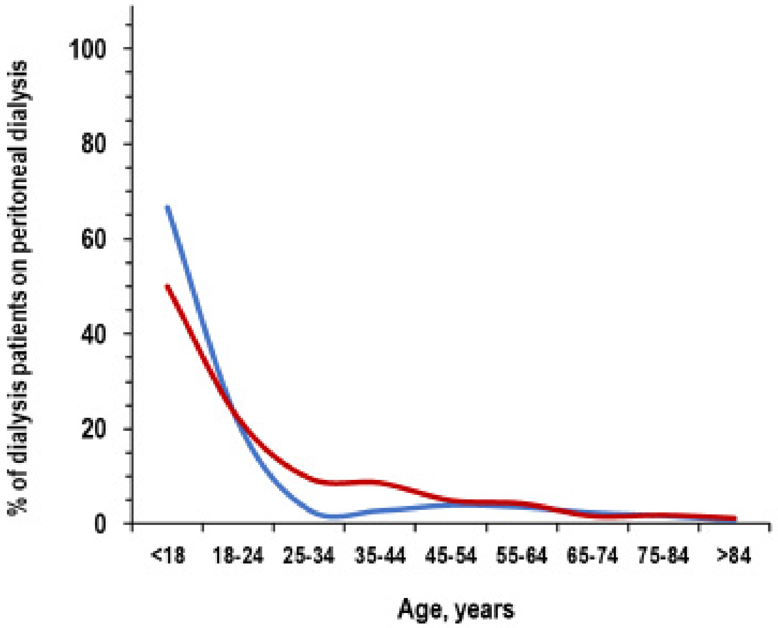

Figure S5 – Incidence of kidney replacement treatment, haemodialysis, peritoneal dialysis, and kidney transplant among residents of Campania region in year 2015: patients per million population (pmp) by age and gender (blue line for males and red line for females, respectively). KRT: kidney replacement treatment

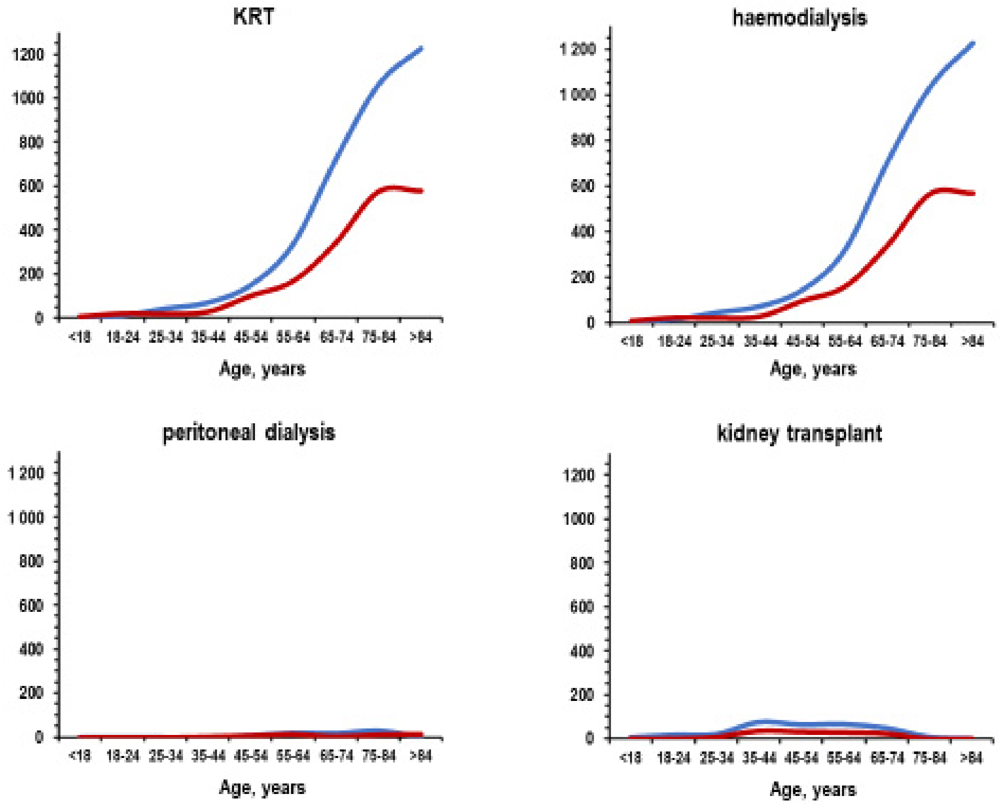

Figure S6 – Incidence of kidney replacement treatment, haemodialysis, peritoneal dialysis, and kidney transplant among residents of Campania region in year 2016: patients per million population (pmp) by age and gender (blue line for males and red line for females, respectively). KRT: kidney replacement treatment

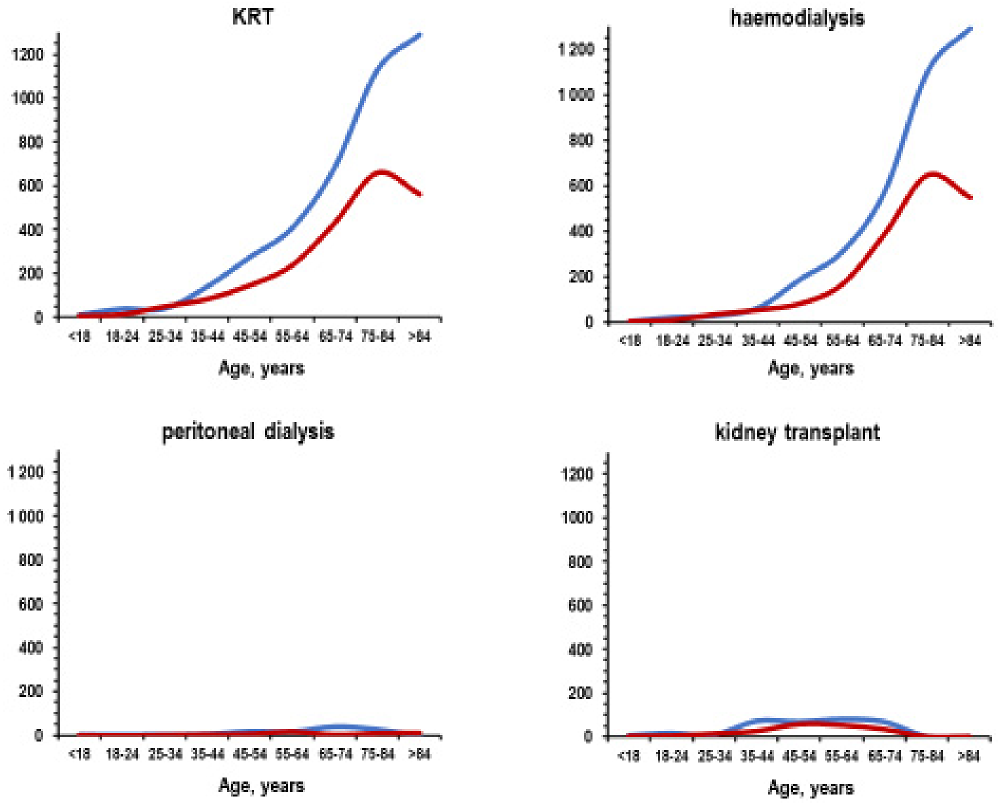

Figure S7 – Incidence of kidney replacement treatment, haemodialysis, peritoneal dialysis, and kidney transplant among residents of Campania region in year 2018: patients per million population (pmp) by age and gender (blue line for males and red line for females, respectively). KRT: kidney replacement treatment

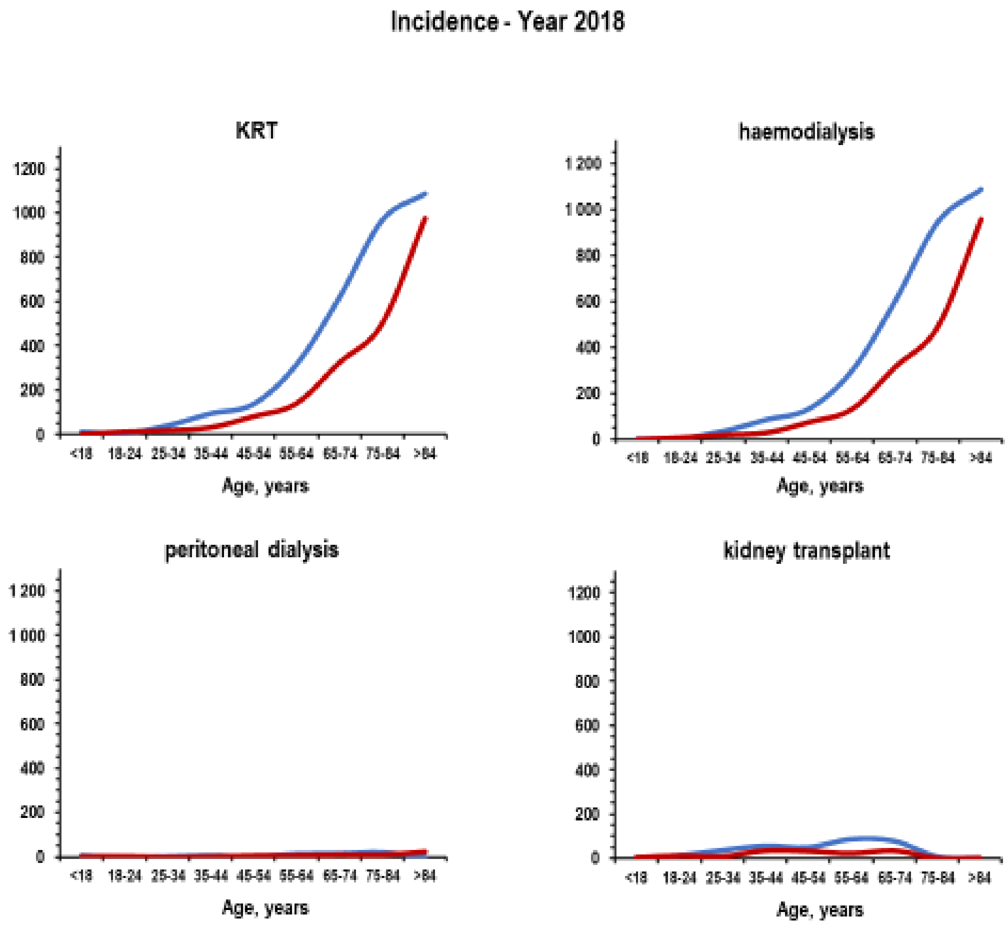

Figure S8 – Overall incidence of kidney transplant from 2015 to 2018 among residents of Campania region on kidney replacement treatment with dialysis: % patients by age at entry in datafile and by gender (blue line for males and red line for females, respectively). KRT: kidney replacement treatment

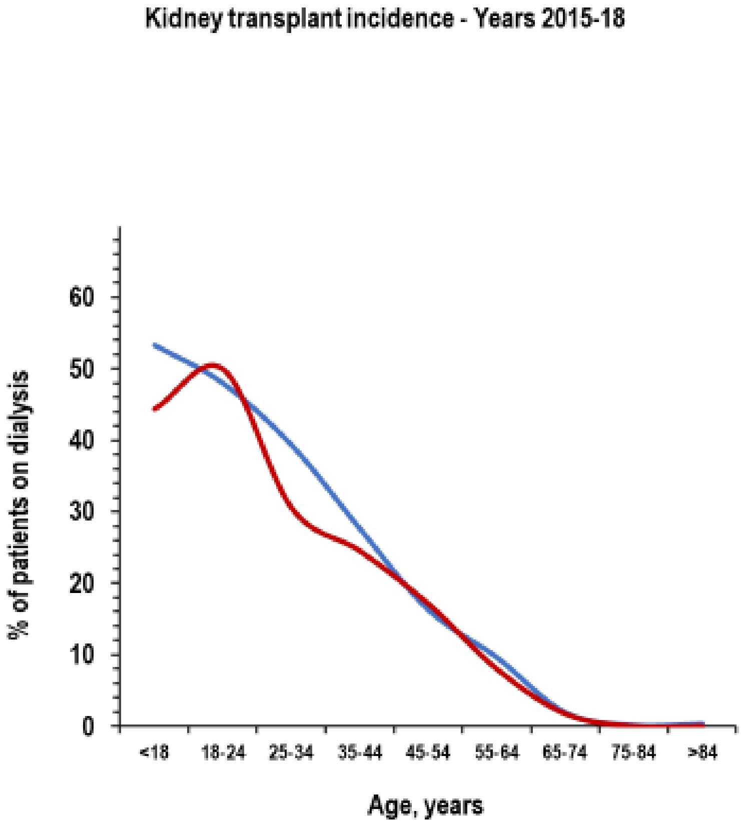

Table S2 – Multi-variate Cox regression analyses on mortality from 2015 to 2018 (dependent variable) by type of kidney replacement treatment among residents in Campania region: HR (*95%CI*), bold character for HRs significantly ≠ 1. KRT: kidney replacement treatment

	Type of KRT		
Independent variables	Haemodialysis<br>N patients = 8236,<br>N events = 3247	Peritoneal dialysis<br>N patients = 237,<br>N events = 73	Kidney transplant<br>N patients = 3240<br>N events = 346
Female gender, y/n=1/0	0.98 *(0.91-1.04)*	0.84 *(0.52-1.34)*	**0.78 *(0.62-0.99)***
Age at entry in datafile, years	**1.05 *(1.05-1.06)***	**1.04 *(1.03-1.05)***	**1.06 *(1.05-1.07)***
Local authority of residence			
Town of Naples	1 (reference)	1 (reference)	1 (reference)
Sub-urban areas of Naples*	**1.17 *(1.07-1.29)***	1.15 *(0.61-2.19)*	0.96 *(0.70-1.31)*
Avellino	1.12 *(0.97-1.29)*	1.40 *(0.60-3.29)*	1.64 *(0.70-1.31)*
Benevento	1.09 *(0.91-1.30)*	1.42 *(0.47-4.26)*	1.15 *(0.65-2.05)*
Caserta	1.11 *(0.99-1.25)*	0.96 *(0.39-2.36)*	1.17 *(0.80-1.71)*
Salerno	0.98 *(0.87-1.10)*	1.50 *(0.77-2.90)*	1.18 *(0.86-1.63)*
De-novo incidence of dialysis, y/n=1/0	**1.12 *(1.04-1.21)***	1.10 *(0.69-1.76)*	0.70 *(0.35-1.41)*

*Sub-urban area of Napoli 2 nord and suburban area of Napoli 3 sud combined together

** incidence from 2015 to 2018 included

Figure S8 – Prevalence of kidney replacement treatment in year 2017: data reported by the Italian Registry of Dialysis and Transplantation for the subgroup of regions with available data in comparison to data of the present study for Campania region: haemodialysis (left panel), peritoneal dialysis (central panel), and kidney transplant (right panel). Data are as patients per million population (pmp) by age (dotted line for Italy and straight line for Campania, respectively). Data of the Italian Registry of Dialysis and Transplantation are from ref. # 7.

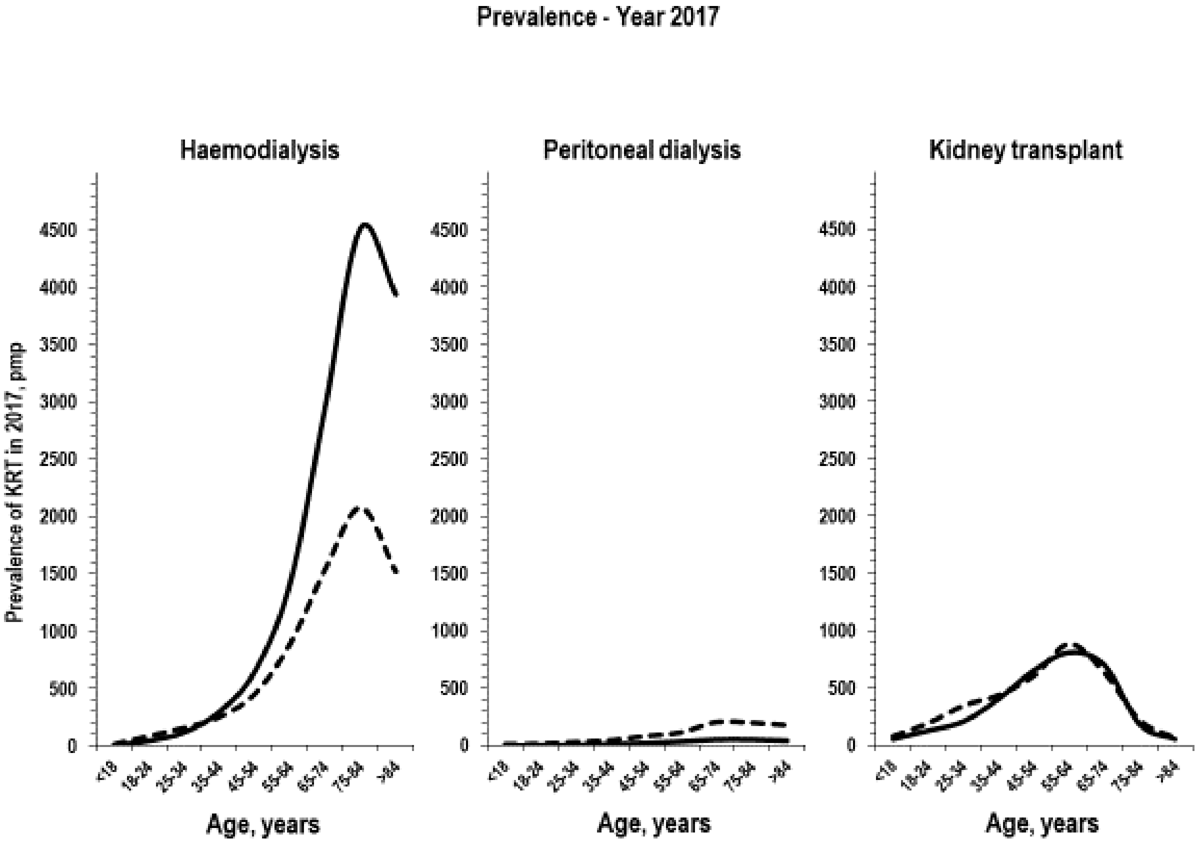

Supplement: Supplementary file 1 [file jcm-10-00449-s001.pdf]
